# Anthropometric and Physiologic Parameters in Cleft Neonates: A Hospital-Based Study

**DOI:** 10.3390/children8100893

**Published:** 2021-10-06

**Authors:** Swati Verma, Falguni Mehta, SukhDev Mishra, Roshan Noor Mohamed, Harshik Kumar A. Parekh, Ramandeep Kaur Sokhi, Anil Kumar Nagarajappa, Mohammad Khursheed Alam

**Affiliations:** 1 Division of Orthodontics and Dentofacial Deformities, Centre for Dental Education and Research, All India Institute of Medical Sciences, New Delhi 110029, India; ramansokhi74@gmail.com; 2 Department of Orthodontics and Dentofacial Orthopaedics, Government Dental College and Hospital, Ahmedabad 380016, Gujarat, India; drfalgunimehta1@gmail.com (F.M.); harshikp@yahoo.com (H.K.A.P.); 3 Department of Bio-Statistics & Data Management, ICMR-National Institute of Occupational Health, Ahmedabad 380016, Gujarat, India; mishra.sukhdev@gmail.com; 4 Department of Pediatric Dentistry, Faculty of Dentistry, Taif University, P.O. Box 11099, Taif 21944, Saudi Arabia; roshan.noor@tudent.edu.sa; 5 Oral Medicine & Radiology, Department of Oral & Maxillofacial Surgery & Diagnostic Sciences, College of Dentistry, Jouf University, Sakaka 72345, Saudi Arabia; dr.anil.kumar@jodent.org; 6 Orthodontic Division, Preventive Dentistry Department, College of Dentistry, Jouf University, Sakaka 72341, Saudi Arabia

**Keywords:** cleft lip and/or palate, neonates, birth weight, birth length, head length, head circumference, maxillary arch dimensions, cleft impression technique, BCLP, UCLP

## Abstract

The oro-facial morphology is greatly affected in neonates with a cleft lip and palate. The initial evaluation of neonate’s body and maxillary arch dimensions is important for treatment planning and predicting growth in cleft patients. The objective of this study was comparative evaluation of the anthropometric and physiologic parameters of cleft and non-cleft neonates in a hospital-based set up. This cross sectional study was conducted on 88 cleft and non-cleft neonates (*n* = 44 in each group) aged between 0 and 30 days after obtaining approval from the institutional ethics committee and positive written informed consent from their parents. Neonates’ body weight, body length, head length, head circumference, and maxillary arch dimensions were measured. Maxillary arch dimensions were measured on dental casts with digital sliding calipers. Statistical analyses performed using the independent *t*-test and one-way ANOVA analysis were followed by Bonferroni correction for post-hoc comparison. The results showed statistically significant differences in birth weight (*p* < 0.0001), head length (*p* < 0.01), head circumference (*p* < 0.007), and maxillary arch dimensions (*p* < 0.0001) between cleft and non-cleft neonates. These findings suggest that cleft neonates had significant anthropometric and physiologic variations than non-cleft neonates.

## 1. Introduction

The cleft lip and/or palate (CL/P) is one of the most common congenital craniofacial abnormality in neonates. The incidence of CL/P is 1.7 per 1000 live births with ethnic and geographical variation worldwide [1] In India, neonates born with craniofacial anomalies comprise about 1.10 per 1000 live births [2]. Mossey et al. reported the incidence of 0.93 per 1000 live births [3]. Another study, in south India, reported the incidence of 1.09 per 1000 live births [4]. The CL/P has a multifactorial etiology that includes both genetic and environmental factors. These environmental risk factors include exposure to tobacco, alcohol, inadequate nutrition intake, infections, and teratogens during 6th to 13th week of intrauterine life [1].

The treatment approach of CL/P in neonates is multidisciplinary. The assessment, diagnosis, and treatment plan starts immediately just after birth. Treatment plan varies from parental psychological support to naso-alveolar molding, cheiloplasty, palatoplasty, orthodontic therapy, and multiple revision surgeries at different stages till adulthood. Correct surgical and non-surgical treatments at the right times are critical for the greatest functional and aesthetic results. In an attempt to assess the general health, as well as the surgical outcome and growth of a cleft neonate at different stages, it is important to determine and maintain the anthropometric and physiological parameters since birth in the first place. For initial evaluation of a neonate’s body, the dimensions of maxillary arch is a crucial factor for treatment planning and growth prediction in cleft patients. To the best of our knowledge, there is a paucity in the literature regarding evaluation of the anthropometric and physiologic parameters of cleft and non-cleft neonates at our tertiary care center. The present cross sectional study was designed as per STROBE guidelines [5] with an objective of comparative evaluation of the anthropometric and physiologic parameters of cleft and non-cleft neonates in a hospital based set up.

## 2. Materials and Methods

### 2.1. Participants

This cross-sectional, hospital-based study was conducted among 88 neonates, 44 with a cleft Lip and/or palate and 44 without (both male and female), aged between 0 and 30 days. Approval from the Institutional Ethics Committee was obtained prior to the initiation of the study (IEC Number: 31 April 2014). The neonates included in the study were divided into two groups: Group I—neonates with a cleft lip and/or palate (experimental group); Group II—age-matched healthy neonates (control group). A positive written informed consent from the parents was taken prior to including neonates for the study. The inclusion criteria of the cleft group included non-syndromic cleft lip and/or palate (unilateral, bilateral, and isolated) neonates aged between birth to 30 days reported to the Department of Orthodontics, Government Dental College, Civil Hospital, Ahmedabad, Gujarat, India between the duration of April 2014 to April 2015. Only neonates with parents who provided written informed consent were considered. The inclusion criteria of the control group were age-matched healthy neonates selected from Department of Paediatrics, Civil Hospital, Ahmedabad, Gujarat, India. The exclusion criteria for both the groups were neonates with preterm birth, systemic abnormality, and associated syndrome, or neonates older 30 days. All 88 neonates in the study were of Gujarati origin. This might be due to the population’s predominance in this geographical area of the country where the study was conducted.

A total of 47 cleft neonates reported at the hospital, 3 of which were excluded from the study due to the presence of associated syndrome. Group I was further divided into three sub groups: Subgroup I—neonates with a unilateral cleft lip and palate (UCLP) (*n* = 22), Subgroup II—neonates with an isolated cleft palate (ICP) (*n* = 10), and Subgroup III—neonates with a bilateral cleft lip and palate (BCLP) (*n* = 12).

### 2.2. Measures

**Figure 1:**
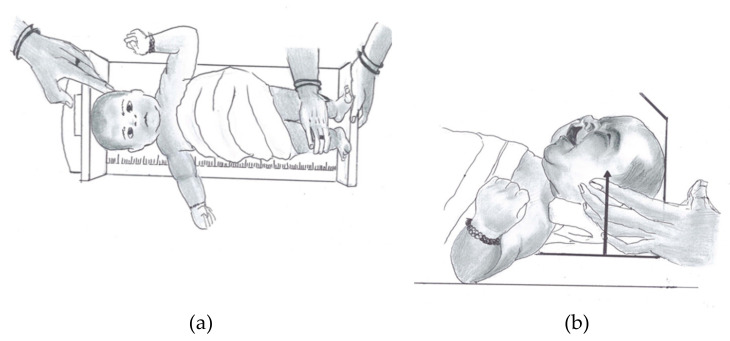
Diagrammatic representation of birth length measurement in neonates. (**a**) At stadiometer; (**b**) Head positioned in the Frankfort vertical plane.

**Figure 2:**
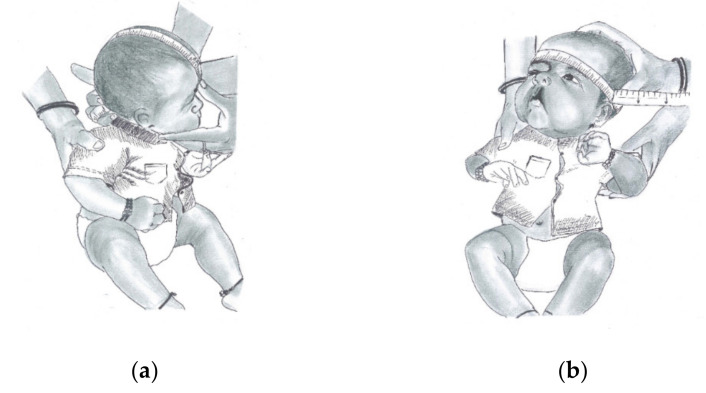
Measurement depiction for (**a**) head length in neonates and (**b**) head circumference in neonates.

The physiological and anthropometric measurements, such as birth weight, birth length, head circumference, head length, and maxillary gum pad dental models (Figure S1), were recorded by an trained and experienced examiner within 48 h of birth, as suggested by Jennson et al. and Cheikh Smile at al. [6,7]. The birth weight (Figure S2) was measured using an electronic digital scale with an accuracy of ± 10 gm. Birth length was measured by horizontal infant stadiometer to the nearest 0.5 cm (Figure 1a,b). Head length (Figure 2a) and circumference (Figure 2b) were measured in supported upright position with a non-extendable flat measuring tape (Table 1).

For taking maxillary arch anthropometric measurements, impression of the neonate’s maxillary gum pad was made within 48 h of birth. Primary impression was taken using impression compound with the neonate in upright position, with back supported in mother’s lap. The cast was poured using a dental plaster, after which a wax spacer was adapted over the plaster model. The next step was to fabricate a perforated, size-compatible special tray using self-cure acrylic material. The final impression was made with elastomeric impression material (putty wash impression) sequentially using heavy body and light body material. The final cast obtained with good details was taken to record linear anthropometric measurements with more accuracy [8,9]. The putty wash impression ensured the good-quality study model [10]. Any residual impression material left in oral cavity was checked by running the pulp of the finger throughout the vestibule and cleft area. The oral cavity was cleaned with sterilized wet gauze piece.

**Figure 3:**
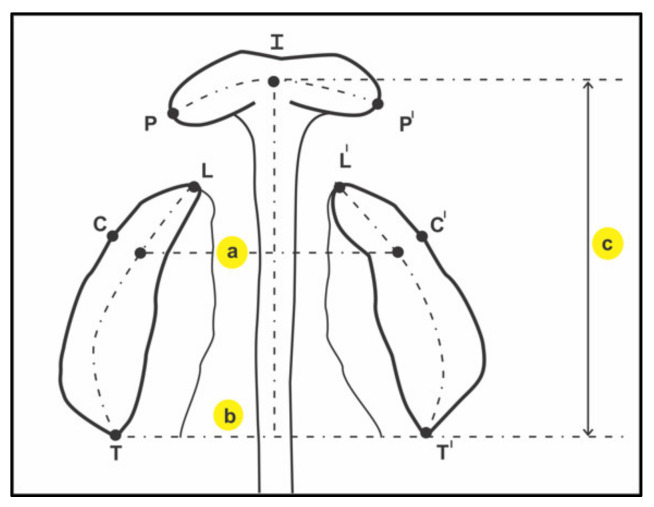
Landmarks for the neonatal cleft maxillary arch, as described by Seckel et al. (1995) [12]. I = incisal point, on the crest of the ridge on the line drawn from the labial frenulum to the incisive papilla; (**a**) C,C′ = canine points, where the lateral sulcus crosses the crest of the ridge; (**b**) T-T′ = tuberosity points, at the junction of crest of the ridge with the outline of the tuberosity; L,L′ = lateral segment margin of cleft, on continuation of the line marking the crest of the ridge; P,P′ = premaxillary margin of cleft, on the continuation of the line marking the crest of the ridge; (**c**) I-TT′ = the perpendicular distance from the incisal point to the T-T′ plane. ‘= denotes the left side (in case of paired landmarks).

The final impression was poured with dental stone. The dental casts with good anatomic details were obtained using this method [9,11]. In the past, materials such as alginate and impression compound were used and their lack of ability to record finer details of cleft were observed, but the chances of tearing and the overflow of material are high [10]. All clinical steps from obtaining cast model of maxillary gum pad till taking the measurements were performed by a single, well-trained, and experienced orthodontist. Airway patency was maintained with caution, overloading of impression material was avoided, and an empathic atmosphere for the parents and guardians was maintained for safety and emotional support. The impression was taken in the clinical setting area that was prepared to handle any inadvertent emergency. The maxillary anthropometric measurements were: inter canine width, inter tuberosity width, arch length, and arch circumference. These were measured as landmarks [12,13], as described in Table 1 using digital sliding calipers measurable to the nearest 0.01 mm. (Figure 3).

### 2.3. Data Analysis

The study data were summarized using descriptive statistics; continuous measurements were given as mean and standard deviation while all categorical data were presented as *n* (%). Summarized data were presented using Tables. The Shapiro–Wilk test was used to check the normality of the data. As the data were found to be normally distributed, bivariate analyses were performed using independent *t*-test and one-way ANOVA analysis, followed by Bonferroni correction for post-hoc comparisons. The level of statistical significance was set at 5% and was denoted as *. Intra-examiner correlation coefficients were assessed using the Kappa co-efficient. The statistical analysis was carried out using Statistical Package for Social Sciences (SPSS) version 21, IBM Inc.

## 3. Results

### 3.1. Sample Demographics

The intra-examiner variability was checked by performing repeat examination on 10% of randomly selected neonates, and then an intra-examiner Kappa coefficient value was found to be 0.82. The mean age of the neonates with cleft were found to be 48 ± 1.17 h and among neonates without cleft it was found to be 36 ± 2.89 h. The descriptive statistics of study sample are shown in Table 2.

### 3.2. Comparison among Cleft and Non-Cleft Neonates

Significant differences were seen in the birth weight, head length, and head circumference of the neonates with and without clefts, i.e., birth weight, head length and head circumference were found greater among neonates without clefts as *p* < 0.05, whereas birth length did not vary among neonates with or without clefts as *p* = 0.337. Inter-canine width, inter-tuberosity width, and arch length were found to be significantly increased among neonates with cleft as *p* < 0.05, whereas arch circumference was found to be significantly higher among neonates without cleft (Table 3).

### 3.3. Comparison of Each Type of Cleft

Among the 44 neonates with a cleft lip and palate, the prevalence of BCLP, ICP, and UCLP was found to be 27.3%, 22.7%, and 50%, respectively (Table 2). No significant differences were seen in the prevalence of BCLP, ICP, and UCLP among males and females (*p* > 0.439) (Table 2). The birth length of the neonates were found to be significantly higher among neonates with BCLP as compared to neonates having ICP and UCLP as *p* = 0.018 whereas the birth weight was found to be almost similar among neonates with ICP, UCLP, and BCLP (Table 4).

The head length was found to be significantly higher among neonates with ICP as compared to the ones with UCLP and BCLP (*p* = 0.019), whereas the head circumference was found to be maximum among neonates with BCLP, marking a significant difference as compared to neonates with ICP (*p* = 0.038). The inter-canine width was found to be significantly greater among neonates with UCLP whereas intertuberosity width, arch length, and arch circumference was seen the highest among neonates with BCLP (*p* < 0.050) (Table 4).

## 4. Discussion

A hospital-based study was conducted on 88 neonates with cleft and non-cleft neonates aged between 0 to 30 days. Neonate’s anthropometric and physiological parameters, birth weight, birth length, head circumference, head length, along with maxillary arch dimensions on dental model were analysed. The standardized methods were followed to record the variables by an experienced operator. Significant differences were seen in the birth weight, head length, and head circumference of the clefts and non-clefts neonates. Birth weight, head length, and head circumference were found to be larger among non-clefts neonates whereas birth length did not vary among the two groups. All recorded maxillary arch anthropometric parameters were found to be statistically significant between the cleft and non-cleft group.

The birth weight is an important physiologic parameter in neonates which reflects the general health of the newly born child. Villar et al. reported that the average birth weight (2.9 ± 0.4 kg) among healthy neonates in India was less than their counterparts in other races, which is in good agreement with our study for non-cleft neonates [14]. Birth weight (2.4 ± 0.5 kg), head length (19.1 ± 4.5 cm) and head circumference (30.8 ± 5 cm) were found significantly decreased in cleft neonates. These findings coincides with the studies by Marques et al., Bowers et al., Felix et al., and Cunningham et al. [15,16,17,18]. Although the fact that Seth and Maxwell demonstrated was that there were no differences between the two groups [19]. No statistically significant differences were found for the birth length (Clefts- 45.0 ± 6.1 cm; Non Clefts 46.02 ± 2.2 cm). This finding is consistent with those of Jensen et al., Duncan et al., Rudman et al., and Ranalli and Mazaheri [6,20,21,22]. Marques et al. found that there is a strong significant correlation between the birth weight, length, and head circumference, and he reported that it was most compromised in cleft neonates in order of birth weight followed by birth length and head circumference [15], which are consistent with our results except for birth length. The etiological factors of the smaller body stature at birth in cleft neonates were proposed by various authors previously [23,24]. These multiple factors can be due to the reduction in sex gonadotropin, anterior pituitary gland function, birth trauma, as well as in genetic, congenital, systematic, and reduced growth hormone prenatally [23,24].

The maxillary arch dimensions recorded in this study between the cleft and non-cleft were inter-canine width, inter-tuberosity width, arch length, and arch circumference. On performing statistical analyses, all of these maxillary arch variables were found significantly different between cleft and non- cleft neonates. Inter-canine width, inter-tuberosity width, and arch length were found to be significantly larger among cleft neonates whereas arch circumference was found to be significantly higher among non- cleft neonates.

The prenatal development of maxilla involves a closely integrated facial and perioral muscle attachment to the underline bone and leads to the formation of complex morphology of the complete palate. Any disruption in the development of the perioral and facial muscle attachment along with the associated skeletal component ultimately affects the dento-alveolar segment morphology. In a complete cleft lip and palate, there is a unilateral or bilateral non-union of palatal process with nasal septum at the prenatal age between 4 to 7 weeks which leads to the development of complete UCLP and BCLP, respectively. ICP is developed between the intrauterine ages of 8 to 12 weeks to non-union of the secondary palate. This creates an imbalance between the perioral musculature. There is an imbalance of forces due to discontinuity in the nasolabiallis insertion, lateral buccinator pull, and other perioral groups of muscles. As result, the anteromedial rotation of the lesser segment and abnormal lateral pull of the greater segment occurs in UCLP. In BCLP, there is an anteromedial collapse of segments bilaterally with protruding the premaxillary complex. Collectively, this leads to increased transverse and anteroposterior dimensions of the maxillary gum pad in CLP neonates [25]. Our findings correlate favorably with the description stated by Markus et al. [25], also confirmed in previous findings by Mello et al. [26], Harila et al. [27], Lo et al. [28], and Honda et al. [14]. The present study is consistent with findings of da Silva et al. [29], who found that maxillary arch dimensions and morphology are distorted by the presence of the cleft.

In this study, the prevalence of BCLP, ICP, and UCLP was found to be 27.3%, 22.7%, and 50%, respectively, within the cleft neonates. Birth length was found to be significantly larger among BCLP neonates as compared to neonates with ICP and UCLP, whereas birth weight was found to be almost similar among three cleft subgroups (Table 4). The head length was found to be significantly larger among ICP neonates as compared to UCLP and BCLP neonates. The head circumference was found to be highest among BCLP neonates, displaying a significant difference with ICP neonates. Inter-canine width was found to be significantly larger among neonates with UCLP (30.8 ±5.4 mm) followed by BCLP (28.70 ± 1.9 mm) and ICP (23.69± 2.1 mm) neonates. These values are in good agreement with Mello et al. [26], Harila et al. [27], and Lo et al. [28], who all stated similar findings. The inter-tuberosity width, arch length, and arch circumference were the largest among neonates with BCLP within the cleft group. This concurs well with Lo et al. [28], and Honda et al. [14]. The dimensions of ICP were closer to the non-cleft group in this study (ICP; ICW 23.69 ± 2.1 mm; ITW 26.50 ± 1.7 mm; AC 53.30 ± 6.7 mm; AL 21.74 ± 2.7 mm).

### 4.1. Clinical Implication

Increased transverse width signifies the lateral displacement and divergence of the palatal shelves in cleft neonates. It may be attributed due to imbalanced forces in the perioral area [28]. The maxillary arch dimensions signifies the amount of tissue deficiency present in cleft neonates. In the present study, larger tissue deficiency was found in UCLP and BCLP. The similar findings in Asian population were suggested previously by Honda et al. [14]. These findings suggest that initial documentation of tissue deficiency may help in the sequential management to minimize scar formation and to provide a positive environment for the growth of maxilla. Although it is multifactorial, the iatrogenic factors can be limited cautiously with the knowledge of these dimensions. The amount of deformity and tissue deficiency helps in treatment planning and decision making to cleft team clinicians. The larger the defect, the more caution that is required for the stability of interventions, such as cheiloplasty, palatoplasty, etc., at different age groups, to plan long-term rehabilitation accordingly. Mutuality and reciprocity between surgeon, clinicians, and health care workers is recommended for good collaboration.

A simple impression technique can provide a true replica of cleft deformity in toto. It is a crucial advantage for maxillary arch assessment at birth in our study [14,30,31,32]. It is cost-effective for the maintenance of initial records for collaborative and decision-making purposes at cleft centers. The other alternatives of dental plaster models used were two dimensional photographs [33] scanned digital models [34,35] and, most recently, intraoral scanners [36,37]. The digital models are beneficial but there is always the added cost of sophisticated desktop and intraoral scanners. A manual measurement of maxillary cast by experienced and trained operators is a viable option to record maintenance in developing countries with poor resources.

### 4.2. Limitation

There are two limitations of our study. The first one is that it was a hospital-based study, and only the cleft neonates who reported to our hospital were recruited in this study. It may not include the neonates who were referred to some other cleft center. However, this center is a centralized tertiary care center so the majority of cleft neonates are referred here for the needful management. The other limitation was the sample size of the cleft subgroups; however, it was a secondary finding of this study. Furthermore, from the results of these subgroups, a clear pattern has emerged regarding the neonates reported to a hospital; this would help in tailoring the individualized presurgical orthopaedic and surgical management with long-term follow-up. In addition, the collected records would help in establishing the baseline data for disease burden and pattern. This could be utilized for hospital administrative purposes by administrators for an efficient regional cleft care program.

## 5. Conclusions

Cleft neonates, compared to non-cleft neonates, had significant anthropometric and physiologic variations.

## Figures and Tables

**Table 1:** Landmarks for variables in the present study [6,7,12].

S. No.	Variables	Units	Definition
1	Head Length (HL)	cm	The maximum length of the head in sagittal plane; measured from glabella, anteriorly to the most prominent point of the head posteriorly.
2	Head Circumference (HC)	cm	The distance recorded from glabella, anteriorly to the most prominent point of the head posteriorly in transverse plane where the measuring tape is anchored and loop around head transversely.
3	Inter-canine width (ICW)	mm	The distance between the canine points C-C′. The canine point is the landmark at intersection of the groove of the lateral labial frenum and the crest of the ridge. (C-C′)
4	Inter-tuberosity width (ITW)	mm	The distance between the tuberosity points T-T′. The tuberosity point is the landmark at intersection maxillary tuberosity and the crest of the ridge outlined on the cast. (T-T′)
5	Arch Length (AL)	mm	A compound measurement. (I-TT′) The perpendicular distance from incisal point to the T-T′ plane.
6	Arch Circumference (AC)	mm	A compound measurement. For UCLP: T-C-I-P+ L-C′-T′ For BCLP: T-C-L + P-I-P′ + L′-C′-T′ For ICP and Controls: T-C-I-C′-T′

**Table 2:** Descriptive Statistics of Study Sample. Age, Sex, Origin.

Variables		Cleft Neonates				Non Cleft Neonates
		UCLP (22)	ICP (10)	BLCP (12)	Total (44)	
Age Mean ± SD		21 ± 1.34	18 ± 1.11	23 ± 1.12	48 ± 1.17	36 ± 2.89
Sex	Female	11 (52.4%)	6 (28.6%)	4 (19.0%)	21	22 (50%)
n (%)	Male	11 (47.8%)	4 (17.4%)	8 (34.8%)	23	22 (50%)
Origin n (%)	Gujarati	22 (50.0%)	10 (22.8%)	12 (27.2%)	44	44 (100%)
	Others	0	0	0	0	-

**Table 3:** Comparison of birth weight, length, head length, head circumference, and maxillary arch dimensions among cleft and non-cleft neonates.

Variables	Group	N	Mean	Std. Deviation	Std. Error Mean	*p* Value
Birth Weight kg	Cleft	44	2.4693	0.53060	0.07999	0.0001 *
	Non-Cleft	44	2.9355	0.41032	0.06186	
Birth Length cm	Cleft	44	45.080	6.1351	0.9249	0.337
	Non-Cleft	44	46.029	2.2322	0.3365	
Head Length cm	Cleft	44	19.148	4.5820	0.6908	0.011 *
	Non-Cleft	44	20.988	1.0134	0.1528	
Head Circumference cm	Cleft	44	30.848	5.0974	0.7685	0.007 *
	Non-Cleft	44	33.042	1.4385	0.2169	
ICW mm	Cleft	44	28.6534	4.97135	0.74946	<0.0001 *
	Non-Cleft	44	21.7686	1.21610	0.18333	
ITW mm	Cleft	44	31.0927	4.86118	0.73285	<0.0001 *
	Non-Cleft	44	27.3818	1.04641	0.15775	
Arch Length mm	Cleft	44	27.4307	7.12700	1.07444	<0.0001 *
	Non-Cleft	44	18.9145	0.66602	0.10041	
Arch Circumference mm	Cleft	44	63.273	13.0836	1.9724	<0.0001 *
	Non-Cleft	44	68.023	1.6352	0.2465	

ICW, Inter-canine width; ITW, Inter-tuberosity width; * Statistical Significance, *p* < 0.05.

**Table 4:** Comparison of birth weight, length, head length, head circumference, and maxillary arch dimensions among different cleft type.

Variable	Cleft Type	N	Mean	Std. Deviation	Std. Error	95% Confidence Interval for Mean		Minimum	Maximum	*p* Value	POST HOC
						Lower Bound	Upper Bound				
Birth Weight kg	UCLP	22	2.4295	0.54416	0.11602	2.1883	2.6708	1.40	3.50	0.525	-
	ICP	10	2.3800	0.66466	0.21019	1.9045	2.8555	1.40	3.50		
	BCLP	12	2.6167	0.37376	0.10790	2.3792	2.8541	2.30	3.40		
Birth Length cm	UCLP	22	43.000	6.8522	1.4609	39.962	46.038	20.0	54.0	0.018 *	3 > 1, 2
	ICP	10	44.850	4.3910	1.3885	41.709	47.991	38.0	53.0		
	BCLP	12	49.083	3.9418	1.1379	46.579	51.588	44.0	55.0		
Head Length cm	UCLP	22	17.705	2.5850	0.5511	16.558	18.851	9.0	24.0	0.019 *	2 > 1, 3
	ICP	10	22.500	7.7172	2.4404	16.979	28.021	15.0	33.5		
	BCLP	12	19.000	2.4863	0.7177	17.420	20.580	17.0	24.0		
Head circumference cm	UCLP	22	31.695	4.3515	0.9277	29.766	33.625	14.0	36.0	0.038 *	2 < 3, 1
	ICP	10	27.300	6.9290	2.1911	22.343	32.257	16.0	32.0		
	BCLP	12	32.250	3.3337	0.9624	30.132	34.368	26.0	36.0		
ICW mm	UCLP	22	30.8782	5.44867	1.16166	28.4624	33.2940	18.00	38.00	<0.0001 *	1 > 3 > 2
	ICP	10	23.6920	2.12724	0.67269	22.1703	25.2137	20.88	26.00		
	BCLP	12	28.7092	1.98762	0.57378	27.4463	29.9720	25.07	32.52		
ITW mm	UCLP	22	32.0845	5.56885	1.18728	29.6155	34.5536	20.00	41.60	<0.0001 *	3, 1 > 2
	ICP	10	26.5050	1.72657	0.54599	25.2699	27.7401	24.00	29.00		
	BCLP	12	33.0975	2.29046	0.66120	31.6422	34.5528	27.82	35.68		
Arch Length mm	UCLP	22	24.6123	2.61028	0.55651	23.4549	25.7696	18.00	30.00	<0.0001 *	3 < 1, 2
	ICP	10	21.7470	2.71107	0.85732	19.8076	23.6864	18.00	28.00		
	BCLP	12	37.3342	5.22381	1.50798	34.0151	40.6532	33.00	48.27		
Arch Circumference mm	UCLP	22	58.545	8.3764	1.7859	54.832	62.259	47.0	77.0	<0.0001 *	1, 2 < 3
	ICP	10	53.300	6.7831	2.1450	48.448	58.152	46.0	68.0		
	BCLP	12	60.750	0.9653	0.2787	60.137	61.363	60.0	62.0		

ICW, Inter-canine width; ITW, Inter-tuberosity width; * Statistical Significance, *p* < 0.05.

## Data Availability

Anonymized data are available on request from the corresponding author and with permission of the participants in the study. The data presented in this study are not publicly available due to ethical reasons (to protect the privacy of participants).

## Supplementary Materials

The following are available online at https://www.mdpi.com/article/10.3390/children8100893/s1, Figure S1: Maxillary Arch Study model. (A) Non-cleft; (B) Unilateral cleft lip and/or palate; (C) Isolated cleft palate; and (D) Bilateral cleft lip and/or palate. Figure S2: Diagrammatic representation of birth weight measurement in neonates.

## References

[1] Mossey P.A., Little J., Munger R.G., Dixon M.J., Shaw W.C. (2009). Cleft lip and palate. Lancet.

[2] Mossey P.A., Munger R., Murray J.C., Shaw W.C., WHO (2002). Reports, Human Genetics Programme: Management of Noncommunicable Diseases: International Collaborative Research on Craniofacial Anomalies. Global Strategies Towards Reducing the Health Care Burden of Craniofacial Anomalies.

[3] Mossey P., Little J. (2009). Addressing the challenges of cleft lip and palate research in India. Indian J. Plast. Surg..

[4] Reddy S.G., Reddy R.R., Bronkhorst E.M., Prasad R., Ettema A.M., Sailer H.F., Bergé S.J. (2010). Incidence of cleft Lip and palate in the state of Andhra Pradesh, South India. Indian J. Plast. Surg..

[5] Vandenbroucke J.P., von Elm E., Altman D.G., Gotzsche P.C., Mulrow C.D., Pocock S.J. (2007). Strengthening the reporting of ob-servational studies in epidemiology (STROBE): Explantion and elaboration. Epidemiology.

[6] Jensen B.L., Dahl E., Kreiborg S. (1983). Longitudinal study of body height, radius length and skeletal maturity in Danish boys with cleft lip and palate. Eur. J. Oral Sci..

[7] Cheikh Ismail L., Knight H.E., Ohuma E.O., Hoch L., Chumlea W.C., International Fetal and Newborn Growth Consortium for the 21st Century (2013). Anthropometric standardisation and quality control protocols for the construction of new, international, fetal and newborn growth standards: The INTERGROWTH-21st Project. BJOG.

[8] Bajaj A., Rao K.S., Sharma S.M., Shetty V. (2011). Modified Presurgical Nasoalveolar Molding in the Infants with Complete Unilateral Cleft Lip and Palate: A Stepwise Approach. J. Maxillofac. Oral Surg..

[9] Patel D., Goyal R., Puri T. (2013). Presurgical Nasoalveolar Moulding—An Adjunct to Facilitate Surgical Repair in Infants with Cleft Lip and Palate. Mod. Plast. Surg..

[10] Marshak B., Assif D., Pilo R. (1990). A controlled putty-wash impression technique. J. Prosthet. Dent..

[11] Grayson B.H., Shetye P.R. (2009). Presurgical nasoalveolar moulding treatment in cleft lip and palate patients. Indian J. Plast. Surg..

[12] Seckel N.G., Van Der Tweel I., Elema G.A., Specken T.F. (1995). Landmark Positioning on Maxilla of Cleft Lip and Palate Infant—A Reality?. Cleft Palate Craniofac. J..

[13] Honda Y., Suzuki A., Ohishi M., Tashiro H. (1995). Longitudinal Study on the Changes of Maxillary Arch Dimensions in Japanese Children with Cleft Lip and/or Palate: Infancy to 4 Years of Age. Cleft Palate Craniofac. J..

[14] Villar J., Ismail L.C., Victora C.G., Ohuma E.O., Bertino E., Altman D.G., Lambert A., Papageorghiou A.T., Carvalho M., Jaffer Y.A. (2014). International standards for newborn weight, length, and head circumference by gestational age and sex: The Newborn Cross-Sectional Study of the INTERGROWTH-21st Project. Lancet.

[15] Marques I.L., Nackashi J.A., Borgo H.C., Martinelli A.P.M.C., Pegoraro-Krook M.I., Williams W.N., Dutka J., Seagle M.B., Souza T.V., Garla L.A. (2009). Longitudinal study of growth of children with unilateral cleft-lip palate from birth to two years of age. Cleft Palate Craniofac. J..

[16] Bowers E.J., Mayro R.F., Whitaker L.A., Pasquariello P.S., LaRossa D., Randall P. (1987). General Body Growth in Children with Clefts of the Lip, Palate, and Craniofacial Structure. Scand. J. Plast. Reconstr. Surg..

[17] Felix-Schollaart B., Hoeksma J.B., Prahl-Andersen B. (1992). Growth Comparison between Children with Cleft Lip and/or Palate and Controls. Cleft Palate Craniofac. J..

[18] Cunningham M.L., Jerome J.T. (1997). Linear growth characteristics of children with cleft lip and palate. J. Pediatr..

[19] Seth A.K., McWilliams B.J. (1988). Weight gain in children with cleft palate from birth to two years. Cleft Palate J..

[20] Duncan P.A., Shapiro L.R., Soley R.L., Turet S.E. (1983). Linear Growth Patterns in Patients With Cleft Lip or Palate or Both. Arch. Pediatr. Adolesc. Med..

[21] Rudman D., Davis G.T., Priest J.H., Patterson J.H., Kutner M.H., Heymsfield S.B., Bethel R.A. (1978). Prevalence of growth hormone deficiency in children with cleft lip or palate. J. Pediatr..

[22] Ranalli D.N., Mazaheri M. (1975). Height-weight growth of cleft children, birth to six years. Cleft Palate J..

[23] Becker M., Svensson H., Källén B. (1998). Birth weight, body length, and cranial circumference in newborns with cleft lip or palate. Cleft Palate Craniofac. J..

[24] Supplement D., Candotto V., Oberti L., Gabrione F., Greco G., Rossi D., Romano M., Mummolo S. (2019). Current concepts on cleft lip and palate etiology. J Biol. Regul. Homeost Agents.

[25] Markus A., Smith W., Delaire J. (1992). Facial balance in cleft lip and palate I. Normal development and cleft palate. Br. J. Oral Maxillofac. Surg..

[26] Mello B.Z.F., Fernandes V.M., Carrara C.F.C., Machado M.A.A.M., Garib D., Oliveira T.M. (2013). Evaluation of the intercanine distance in newborns with cleft lip and palate using 3D digital casts. J. Appl. Oral Sci..

[27] Harila V., Ylikontiola L.P., Palola R., Sándor G.K. (2013). Maxillary arch dimensions in cleft infants in Northern Finland. Acta Odontol. Scand..

[28] Lo L.-J., Wong F.-H., Chen Y.-R., Lin W.-Y., Ko E.W.-C. (2003). Palatal Surface Area Measurement: Comparisons among Different Cleft Types. Ann. Plast. Surg..

[29] da Silva Filho O.G., Ramos A.L., Abdo R.C. (1992). The influence of unilateral cleft lip and palate on maxillary dental arch morphology. Angle Orthod..

[30] Brief J., Behle J., Stellzig-Eisenhauer A., Hassfeld S. (2005). Precision of Landmark Positioning on Digitized Models from Patients with Cleft Lip and Palate. Cleft Palate Craniofac. J..

[31] Darvann T.A., Hermann N.V., Ersbøll B.K., Kreiborg S., Berkowitz S. (2007). Palatal Surface Area of Maxillary Plaster Casts—A Comparison between Two-Dimensional and Three-Dimensional Measurements. Cleft Palate Craniofac. J..

[32] Long R.E., Hathaway R., Daskalogiannakis J., Mercado A., Russell K., Cohen M., Semb G., Shaw W. (2011). The Americleft study: An inter-center study of treatment outcomes for patients with unilateral cleft lip and palate part 1. Principles and study design. Cleft Palate Craniofac. J..

[33] Leenarts C., Bartzela T., Bronkhorst E., Semb G., Shaw W., Katsaros C., Kuijpers-Jagtman A. (2012). Photographs of dental casts or digital models: Rating dental arch relationships in bilateral cleft lip and palate. Int. J. Oral Maxillofac. Surg..

[34] Neuschulz J., Schaefer I., Scheer M., Christ H., Braumann B. (2013). Maxillary reaction patterns identified by three-dimensional analysis of casts from infants with unilateral cleft lip and palate. J. Orofac. Orthop..

[35] Asquith J., McIntyre G. (2012). Dental Arch Relationships on Three-Dimensional Digital Study Models and Conventional Plaster Study Models for Patients with Unilateral Cleft Lip and Palate. Cleft Palate Craniofac. J..

[36] Chaudhari P.K., Kharbanda O.P. (2017). Intraoral 3D Scanning in Cleft Care. Cleft Palate Craniofac. J..

[37] Batra P., Gribel B.F., Abhinav B.A., Arora A., Raghavan S. (2019). OrthoAligner “NAM”: A Case Series of Presurgical Infant Orthopedics (PSIO) Using Clear Aligners. Cleft Palate Craniofac. J..

