# Supplementary material for: Anthropometric and Physiologic Parameters in Cleft Neonates: A Hospital-Based Study
Source: Children (Basel). 2021 Oct 6;8(10):893. doi: 10.3390/children8100893 (PMC8534988; doi:10.3390/children8100893)
Supplement: Supplementary file 1 [file children-08-00893-s001.zip › children-1367297-SI.pdf]

Supplementary

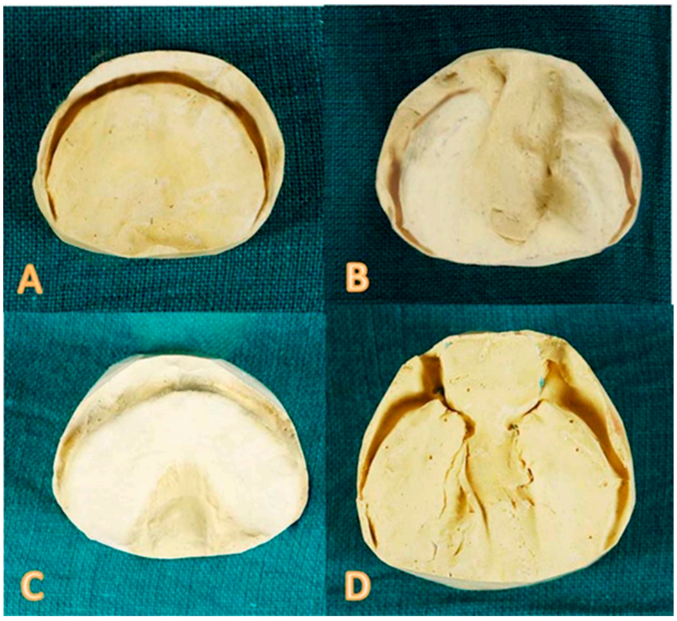

Figure S1. Maxillary Arch Study model. (**A**) Non-cleft; (**B**) Unilateral cleft lip and/or palate; (**C**) Isolated cleft palate; and (**D**) Bilateral cleft lip and/or palate.

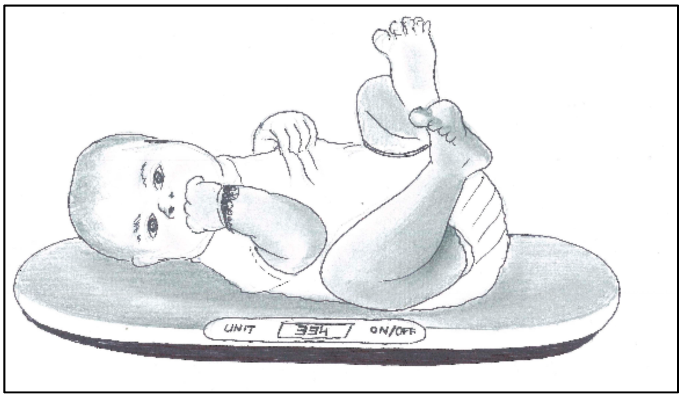

Figure S2. Diagrammatic representation of birth weight measurement in neonates.
